# Divergent Approaches to Virulence in *C. albicans* and *C. glabrata*: Two Sides of the Same Coin

**DOI:** 10.3390/ijms20092345

**Published:** 2019-05-11

**Authors:** Mónica Galocha, Pedro Pais, Mafalda Cavalheiro, Diana Pereira, Romeu Viana, Miguel C. Teixeira

**Affiliations:** 1 Department of Bioengineering, Instituto Superior Técnico, Universidade de Lisboa, 1049-001 Lisboa, Portugal; monicagalocha@gmail.com (M.G.); pedrohpais@tecnico.ulisboa.pt (P.P.); mafalda.cavalheiro@tecnico.ulisboa.pt (M.C.); diana.pereira@tecnico.ulisboa.pt (D.P.); romeuviana@outlook.com (R.V.); 2 iBB-Institute for Bioengineering and Biosciences, Biological Sciences Research Group, Instituto Superior Técnico, University of Lisbon, Av. Rovisco Pais, 1049-001 Lisboa, Portugal

**Keywords:** *Candida*, host-pathogen interaction, virulence, biofilm formation, morphology, immune evasion

## Abstract

*Candida albicans* and *Candida glabrata* are the two most prevalent etiologic agents of candidiasis worldwide. Although both are recognized as pathogenic, their choice of virulence traits is highly divergent. Indeed, it appears that these different approaches to fungal virulence may be equally successful in causing human candidiasis. In this review, the virulence mechanisms employed by *C. albicans* and *C. glabrata* are analyzed, with emphasis on the differences between the two systems. Pathogenesis features considered in this paper include dimorphic growth, secreted enzymes and signaling molecules, and stress resistance mechanisms. The consequences of these traits in tissue invasion, biofilm formation, immune system evasion, and macrophage escape, in a species dependent manner, are discussed. This review highlights the observation that *C. albicans* and *C. glabrata* follow different paths leading to a similar outcome. It also highlights the lack of knowledge on some of the specific mechanisms underlying *C. glabrata* pathogenesis, which deserve future scrutiny.

## 1. Introduction

Infections caused by fungi affect millions of people worldwide, with the overall mortality rate estimated to be roughly 1,350,000 deaths per year [1]. Among pathogenic fungi, *Candida* species are responsible for the most common invasive fungal disease in developed countries—the candidiasis [2]. *Candida* species live as commensals on mucosal surfaces where they are constituents of the normal microbiota of the oral cavity and gastrointestinal and vaginal tracts. However, they can opportunistically become pathogenic under suitable conditions, such as host-disrupted microbiota or immunocompromised hosts, being responsible for clinical manifestations from mucocutaneous overgrowth to bloodstream infections [3,4,5]. Of the various *Candida* species, *Candida albicans* and *Candida glabrata* not only account for 60% of *Candida* species present in the human body, but also constitute the most prevalent of the pathogenic *Candida* species, being responsible for more than 400,000 life-threatening infections worldwide every year [3,6]. 

*C. albicans* and *C. glabrata* are the two most common pathogenic yeasts of humans, yet they are phylogenetically, genetically, and phenotypically very different. On one hand, *C. albicans* diploid genome carries several gene families that are associated with virulence [7]. These include the ALS (agglutinin-like sequence) adhesins, required for host adhesion, secreted aspartyl proteases (SAPs) and phospholipases, which allow for the degradation of host barriers and the invasion of surrounding tissue, and proteins involved in oligopeptide and iron transfer [8,9,10]. On the other hand, *C. glabrata* mechanisms of tissue invasion are mostly unknown, although it is hypothesized to possibly occur by endocytosis induction of host cells [11]. Its haploid genome encodes a large group of glycosylphosphatidylinositol (GPI)-anchored cell wall proteins, such as the adhesins from the *EPA* gene family, implicated in fungus–host interactions or biofilm formation, and a family of aspartic proteases (yapsins) which are mainly associated with cell wall remodeling and possible immune evasion [12,13]. Moreover, key virulence attributes of *C. albicans,* which are known to be the basis of its pathogenicity, are absent in *C. glabrata* [14]. Switching from yeast to hyphal growth not only allows consistent biofilm production but also enables *C. albicans* to be highly invasive and escape macrophage engulfment [15,16,17,18,19]. Nevertheless, both species are known to use biofilms to colonize the surface of several medical devices based on different materials [20]. Unlike *C. albicans*, it has been demonstrated that *C. glabrata* lets itself be taken up by macrophages, where it persists and divides for long periods of time eventually leading to cell lysis due to fungal load [21,22]. It has the ability of detoxifying oxidative radical species and disrupting normal phagosomal maturation, leading to the inhibition of phagolysosome formation and phagosome acidification [21,23]. 

The interaction between *Candida* and its host cells is characterized by a complex interplay between the expression of fungal virulence factors and the host immune system, and the presence of other microorganisms affects this interplay. This review aims to explore and compare the remarkably distinct paths toward virulence trailed by the two most common causative agents of candidiasis worldwide. On one hand, *C. albicans* is known for its ability to evade host defenses and form bulk biofilms due to its ability to undergo filamentous growth, while on the other hand, *C. glabrata* is an unusually stress-tolerant organism able to survive and replicate inside the immune system cells. Despite having such distinct virulence features, *C. glabrata* and *C. albicans* are frequently co-isolated [11].

## 2. Host Damage and Invasion

There is a variety of defense mechanisms through which the human host is able to prevent invasion by pathogenic microorganisms, such as *C. albicans* and *C. glabrata*. These mechanisms consist not only of physical but also of chemical barriers. For instance, epithelial cells, which in most cases are the first line of contact between host and pathogen, function as the prime physical barrier restraining *Candida* from invasion of the underlying tissue. On one hand, these cells are interconnected through “tight junctions” preventing the entry of microorganisms into interepithelial space and eventually into the bloodstream [24,25]. On the other hand, some types of epithelial cells, such as those in the intestinal or vaginal epithelium, are able to produce a mucus layer by secreting mucins [24,26]. This layer impairs *Candida* invasion by preventing contact with the epithelium surface. Likewise, in the oral cavity the flow of saliva plays an important role as both a physical and a chemical barrier as it not only prevents the adhesion to mucosa and dental surfaces but also contains several antimicrobial agents that impair the contact of *Candida* with the oral epithelium [27,28]. Another chemical barrier against *Candida* establishment is the presence of gastric acid and bile in the digestive system which creates a harsh environment for fungal growth. Nevertheless, these human pathogens are known to have a remarkable ability to adapt to these adverse conditions and proliferate. 

**Figure 1:**
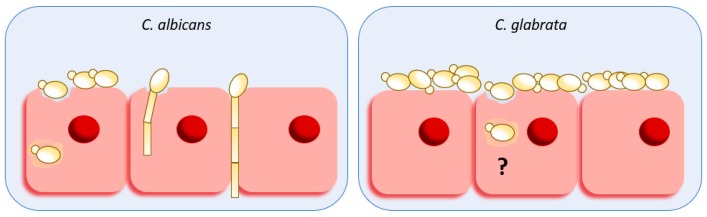
Schematic representation of *C. albicans* and *C. glabrata* host damage and invasion. *C. albicans* enter host cells through induced endocytosis or by active penetration (inter- and intra-cellular) of hyphal forms associated with the release of hydrolytic enzymes, resulting in the damage of cells and loss of epithelial integrity. Induced endocytosis of host cells is thought to be the mechanism behind *C. glabrata* tissue invasion.

*C. albicans* relies on two distinct invasion mechanisms to gain entry into host cells: (i) induced endocytosis and (ii) active penetration of hyphal forms through physical forces of hyphae production associated with lytic enzyme secretion [29] (Figure 1). Nonetheless, depending on the host cell, these invasion mechanisms are thought to be exploited to a different extent. For instance, it was demonstrated that while invasion into oral epithelial cells occurs via both routes, invasion into intestinal epithelial cells occurs only via active penetration under normal conditions [29,30]. 

Living within host cells is a profitable strategy since it enables cells to deal with the host immune system and antimicrobial treatment, and there are plenty of nutrients available and an absence of competition with other commensal microorganisms. Additionally, dissemination into deeper tissues and ultimately into the bloodstream is easier from within the cell. Fungal invasion via induced endocytosis is dependent on dynamic microfilaments of the host. In *C. albicans*, the GPI-anchored hypha-associated protein Als3 interacts with mammalian cadherins, mimicking the establishment of adherence junctions. This process leads to rearrangements in the actin cytoskeleton that ultimately lead to fungal cell internalization [31]. Similarly, Ssa1, a member of the HPS70 heat shock protein family, was reported to play the same role as Als3, being essential for maximal host cell damage and subsequently fungal cell endocytosis [32]. However, *EED1* was the first fungal gene reported as being required for epithelial escape and interepithelial dissemination and not for initial invasion into epithelial cells [33]. Moreover, it is thought that the contact with the epithelium, among several other stimuli, is a highly potent inducer of *C. albicans* filamentation. In turn, the contact between hyphal cells and epithelial cells induces host defense mechanisms such as the formation of epithelial cell protrusions surrounding the hyphae, and membrane ruffling, which is characteristic for induced endocytosis [34]. 

The ability to switch between yeast and hyphal growth forms is one of the most discussed and best-investigated virulence attributes of *C. albicans*. This morphology switch is activated by well-established kinase-based signal transduction pathways and is triggered by diverse host environmental cues, including temperature, pH, serum, and CO_2_ and is linked to several steps during host invasion [35,36]. The extracellular signals are transmitted via Ras to both protein kinase A and the MAP kinase cascade [37,38,39], inducing hyphal differentiation through the activation of a number of transcription factors such as Efg1 [40] and Ume6 [41]. The transcriptional repressor Nrg1 is inactivated and removed from the hyphal-specific gene promoters, thereby allowing the induction of hyphal morphogenesis [42,43]. 

Invasion into epithelial cells via active penetration relies on a combination of physical pressure employed by the growing hyphae and the secretion of hydrolytic enzymes. Moreover, hyphal cells are capable of directional growth in response to contact with a solid surface (thigmotropism) which enables *C. albicans* to specifically identify and invade intercellular junctions, thereby damaging the epithelium compact structure [44]. Interestingly, it was very recently discovered that *C. albicans* release hydroxyphenylacetic acid (HPA) during hyphal growth [45]. Its production seems to occur through the same pathway and the same precursors as tyrosol, which is able to stimulate hypha induction in *C. albicans* [46], therefore their biological functions are likely to be the same. Additionally, along with the active penetration by hyphal cells, there is also the secretion of hydrolytic enzymes, such as SAPs and phospholipases, which can digest epithelial cell surface components enabling the entrance into or between host cells [47]. SAPs are the best-characterized members of the *C. albicans* hydrolytic enzymes, as is well reviewed by Hube and Naglik [48]. Interestingly, these tissue damaging enzymes not only have distinct optimal pH requirements but also are growth stage and infection site related [47]. For instance, *SAP1–SAP3* are yeast growth-associated and have optimum activity at lower pH values, while *SAP4–SAP6* are hyphal growth-associated and have optimum activity at higher pH values [49]. Similarly, it was demonstrated that *SAP1, SAP3,* and *SAP8* are preferentially expressed in vaginal, rather than oral, *C. albicans* infections [47]. One possible explanation for the existence of a significant number of different *SAP* genes may be the necessity for specific and optimized proteinases during the different stages of an infection [48]. Additionally, the cytolytic peptide toxin of *C. albicans* candidalysin, encoded by the hypha-associated gene *ECE1*, was recently found to be essential for damage of enterocytes and is a key factor in subsequent fungal translocation, suggesting that transcellular translocation of *C. albicans* through intestinal layers is mediated by candidalysin [25]. Moreover, phytase activity in *C. albicans* was demonstrated to be important for virulence. Phytate is a major storage form of phosphorus in plants and is abundant in the human diet and intestinal tract [50]. Recently, it was reported that decreased phytase activity leads to a reduced ability to form hyphae, attenuated in vitro adhesion, and reduced ability to penetrate human epithelium [51]. 

While the transition from yeast to hyphae has been extensively studied in *C. albicans*, the switch from hyphae to yeast still remains poorly understood [35]. Nevertheless, both yeast and hyphae forms are found in infection sites, which suggests that both forms are implicit in the infection process. Interestingly, it was demonstrated that depending on the infected organ, one or the other morphology predominates [52]. It is thought that the yeast form is important for dissemination upon infection, whereas hyphae forms are more relevant to attachment, host invasion, and tissue damage [53].

Unlike *C. albicans*, and despite the existence of some reports demonstrating that *C. glabrata* forms pseudohyphae [54,55], the pathogenicity of *C. glabrata* seems to be independent of morphology. The most common route for this pathogen to reach the bloodstream is through the iatrogenic breach of natural barriers, such as the use of catheters, trauma, or surgery. 

In 2000, Csank and Haynes [54] reported for the first time that *C. glabrata* can undergo morphological change and grow as a pseudohyphae on solid nitrogen starvation media. This invasive growth mode could be a possible mechanism of host invasion, however, this phenomenon has not yet been reported in vivo. Despite lacking this prime virulence feature, this opportunistic pathogen is still able to reach the human bloodstream and cause infection. In some cases, *C. glabrata* can involuntarily reach the bloodstream through nosocomial conditions, namely surgery, catheter, parenteral nutrition, and burn injury [56]. However, even when these external factors are abrogated, *C. glabrata* is able to invade the host and colonize different tissues, as shown in an intragastrointestinal mouse model of infection [57] and in a chorioallantoic membrane chicken embryo model of infection [58]. Thus, *C. glabrata* must have other invasion mechanisms.

Co-infection with other microorganisms may be a possible explanation to the invasive capacity of *C. glabrata* since this yeast is often co-isolated in infections with *C. albicans* [59,60,61] or even other pathogens such as *Clostridium difficile* [62]. In the co-infected environment, *C. glabrata* cells may exploit the tissue invasion and destruction caused by *C. albicans* to access nutrients and reach the bloodstream. In fact, Tati et al. (2016) [63] demonstrated that when mice are infected with *C. glabrata* alone, oropharyngeal candidiasis is negligible, however, when co-infected with *C. albicans*, an increased colonization by *C. glabrata* was observed. This effect was attributed to the binding of *C. glabrata* to *C. albicans* hyphae [63] and similar results were reported by Alves et al. (2014) [64] using a reconstituted human vaginal epithelium. Nonetheless, the intracytoplasmic presence of *C. glabrata* was detected in oral epithelial cells [65] and vaginal epithelial cells [66] and it has been shown that when endocytosis is inhibited, the internalization of *C. glabrata* is prevented [65]. This suggests that induced endocytosis by host cells could be the most likely mechanism of *C. glabrata* internalization (Figure 1). 

As referred to before, the tissue/cell damaging ability of *C. glabrata* is lower compared to *C. albicans*. In *C. albicans*, secreted hydrolytic enzymes are considered to be important destructive factors that damage host tissues, providing nutrients for its propagation. However, the production of these hydrolytic enzymes is very low or even null in *C. glabrata,* wherefore its importance for virulence does not seem to be as relevant as it is in *C. albicans* [67,68,69,70,71,72,73]. The proteinase enzyme is responsible for protein degradation resulting in tissue invasion. Among the proteases, SAPs are considered crucial for the pathogenicity of *C. albicans* [74]. Although *C. glabrata* does not express SAPs [75], its genome contains 11 non-secreted GPI-linked aspartyl proteases (*YPS* genes), which are surface-exposed aspartic proteases required for virulence, known as yapsins [13]. These yapsins are important for cell wall maintenance, remodeling, cell to cell interactions, and resistance to cell wall stress, however, their direct link with virulence is not very well characterized yet. Otherwise, phospholipases promote the destruction of cell membrane phospholipids, causing cell damage and lysis which allows a greater invasive capacity [76]. Some of the *C. glabrata* strains are able to produce these enzymes [67,68,70,71,77], which appear to play a role in *C. glabrata*-associated persistent candidemia [9]. However, phospholipase production in *C. glabrata* is lower than in *C. albicans,* and in some cases inexistent [69,72,73], therefore its relation to *C. glabrata* virulence is not clear and needs further analysis. 

## 3. Adhesion and Biofilm Formation

The ability to infect and prevail in the human host is related to different pathogenesis factors, of which biofilm formation excels [19,78,79,80]. *Candida* species ability to form biofilms on medical devices increases mortality rates associated with infections, while often forcing the treatment to include the removal of the medical device [81]. A lot of efforts have been put into understanding the molecular basis of *Candida* species biofilm formation [20].

Adhesion is one of the most relevant and advantageous capacities of the yeast cell wall. It allows cells to colonize mucosal surfaces and prevail in a nutritional environment, being the first critical step for biofilm formation, which serves as a shield against adverse conditions, as well as a highly drug-resistant reservoir of infective cells [82,83]. *C. albicans* and *C. glabrata* pathogenesis has been strongly related to adhesion, which is considered a crucial virulence factor in these yeasts [84,85,86]. In this regard, both *Candida* species are able to not only attach to mammalian host cells (epithelial, endothelial, and immune cells) but also to other microbes (bacteria and other *Candida* species) and abiotic surfaces, such as medical devices [85,87]. Several studies have tried to understand the nature of adherence to plastic surfaces. For instance, cell surface hydrophobicity (CSH) seems to have a positive correlation with adhesion in both *C. albicans* and *C. glabrata* species, thus, adhesion is mediated by van der Waals forces. Moreover, compared to *C. albicans*, the relative CSH of *C. glabrata* has been shown to be significantly higher [88,89,90].

Both *Candida* species have a set of proteins that enable attachment, known as adhesins. The most important *C. albicans* adhesins are agglutinin-like sequence (Als) proteins (Als1–7 and Als9) [91] and hypha-associated GPI-linked protein (Hwp1), known to be required for adhesion and virulence in vivo, and also being associated to biofilm formation through interaction with Als1 and Als3 adhesins [92,93,94]. Other adhesins required for adhesion and biofilm formation include Hwp2 [95] and Eap1 [96,97,98]. *C. glabrata* expresses a large group of adhesins, belonging to the epithelial adhesin (Epa) family, which is encoded by 17 to 23 genes, depending on the strain [78]. Among these, Epa1 is a major virulence player in *C. glabrata*, and mediates 95% of in vitro adhesion to epithelial cells [99]. This adhesin is highly heterogeneous among *C. glabrata* clinical isolates, being an important virulence factor [100]. Epa6 and Epa7 are involved in kidney and bladder colonization in vivo and boost biofilm formation [101,102,103,104]. Transcriptomic and proteomic studies have revealed that besides *EPA* genes, *C. glabrata* holds other biofilm-related adhesin families, such as Pwp (encoded by seven members *PWP1–7*), Aed (*AED1* and *AED2*), and Awp (encoded by 12 members *AWP1–6* and *AWP8–13*), which are usually found in significantly higher numbers in clinical isolates, consistently with an important role in pathogenesis [12,105,106,107].

The successful pathogenicity of these yeasts relies on its flexibility, which allows for adaptation and proliferation under both nutrient-rich and nutrient-poor conditions. Several studies have reported the importance of host and antifungal selective pressure on virulence traits as adhesion [108,109]. A recent study conducted by Vale-Silva et al. (2017) [110] used the PacBio technology to compare the genomes of two sequential *C. glabrata* clinical isolates and observed a significant increase in the number of adhesin-encoding genes (101 and 107) when compared to the CBS138 genome (63), despite the limited variation between the two studied isolates. The same authors, along with Ni et al. (2018) [111], further linked the increased expression of the adhesin gene *EPA1* with gain-of-function (GOF) mutations in the *PDR1* gene in drug-resistant clinical isolates [112]. *EPA1* has been strongly related to adhesion of *C. glabrata* cells to mammalian epithelial cells both in vitro [99] and in vivo [113]. Furthermore, Salazar et al. (2018) [114] also observed a GOF mutation in the *PDR1* gene, which led to changes in the transcriptome when compared to the CBS138 strain. Among the genes identified as having the highest number of non-synonymous SNPs, there were several genes encoding adhesins and, agreeing with Vale-Silva et al. (2013) [115], the number of adhesin-expressed genes varied when compared to other GOF *PDR1* mutations [114]. This reinforces the idea that antifungal treatment deploys a tight selective pressure which results in changes at the genomic and transcriptional levels, particularly affecting adhesin-encoding genes [116]. The transcription factor Cst6 was found to also play a role in *C. glabrata* adhesion and biofilm formation, negatively regulating the expression of *EPA6* [103]. In *C. albicans*, a transcriptional regulatory network comprising nine regulators (Bcr1, Brg1, Efg1, Flo8, Gal4, Ndt80, Rob1, Rfx2, and Tec1) was identified in in vitro and in vivo studies as underlying the biofilm formation phenomenon in this pathogenic yeast [117,118,119,120,121].

Hyphae formation, which is exclusive to *C. albicans*, when compared to *C. glabrata* is also an important trait in biofilm development. Various studies have described hyphae as exhibiting improved adhesion to the human epithelium, with these cells displaying increased expression of Als1, Als3, and Hwp1 [122,123,124,125]. Interestingly, Tati et al. (2016) [63] characterized the co-colonization of *C. glabrata* and *C. albicans* in a murine model of oropharyngeal candidiasis (OPC) and demonstrated that this is driven by specific adhesins in both species. Namely, the *C. albicans* Als3 and Als1 adhesins are crucial for in vitro binding of *C. glabrata* cells to *C. albicans* hyphae and for further in vivo establishment of infection. Considering *C. glabrata* cells, incubation with *C. albicans* hyphae led to the overexpression of the adhesins *EPA8*, *EPA19*, *AWP2*, *AWP7,* and *CAGL0F00181* [63].

**Figure 2:**
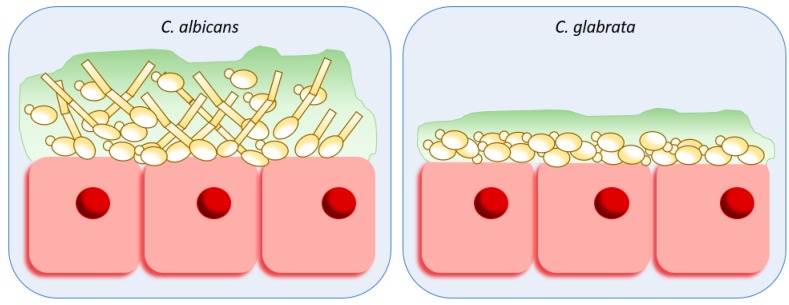
Schematic representation of *C. albicans* and *C. glabrata* biofilm formation. *C. albicans* forms thicker biofilms, with much more biomass in the end of biofilm formation and produces more extracellular matrix than *C. glabrata*. *C. albicans* mature biofilms are composed by a dense network of pseudohyphae, hyphae, and yeast cells, whereas *C. glabrata* biofilms are composed by compact yeast cells only, forming a thin but dense biofilm.

After adhesion, *Candida* species are known to be able to form a 3D-structure of cells embedded in a gel-like matrix [20,126,127]. In order to achieve this, surface-adhered cells begin to adhere to other *Candida* cells, initiating the formation of discrete colonies, which correspond to an early phase of biofilm formation. At this point, the intermediate phase begins with the cellular production and secretion of important molecules, known as extracellular polymeric substances (EPS), that will constitute the extracellular matrix of the biofilm, protecting the cells and ensuring a more developed 3D-structure. The final structure is reached after the maturation phase, where more cells and the matrix are originated. Mature biofilms might also suffer the detachment of some cells that can spread to form new biofilms on other niches of the host, a process called the dispersal phase [20,126,128,129]. Although this process is true for every *Candida* species, there are differences between *C. albicans* and *C. glabrata* biofilms, regarding their dimensions and structure, cell morphology, EPS produced and secreted, response to environmental cues, and resistance to antifungal drugs (Figure 2).

A very clear difference between *C. albicans* and *C. glabrata* mature biofilms is the dimension and total biomass of each biofilm. *C. glabrata* in vivo biofilm formation leads to a thickness of 75–90 ± 5 µm, which is half of the normal thickness of *C. albicans* biofilms [130], with much less biomass in the end of biofilm formation compared to *C. albicans* [131]. The organization of the biofilm structure also differs between these two species. *C. albicans* biofilms are arranged in a three-dimensional structure with different morphologies and empty spaces between cells [132], where microchannels are formed [133]. On the other hand, *C. glabrata* biofilms are thinner, but display a higher density of cells, tightly packed together [132]. Although *C. glabrata*’s biofilms are composed by yeast cells only [131,132], the same is not true for *C. albicans* biofilms, where different morphologies arise. *C. albicans* mature biofilms are composed by a dense network of pseudohyphae, hyphae, and yeast cells [134]. This filamentation process in biofilm formation is controlled by the transcription factor Efg1, without which *C. albicans* only forms scarce monolayers of elongated yeast cells on polyurethane catheters and polystyrene [135,136]. 

Although *C. albicans* produces more extracellular matrix than *C. glabrata* [137], the main components of the matrix, proteins, and carbohydrates, are the same in both biofilms [131,138,139,140]. Interestingly, *C. glabrata* has a very high content of proteins and carbohydrates, which is five times higher than that found on the biofilms of other non-*albicans Candida* species [131]. *C. albicans’* matrix is also composed by other lipids (mainly neutral glycerolipids, polar glycerolipids, and a small percentage of sphingolipids) [139], phosphorus, and uronic acid [140]. Biofilms of both species also have a small content of extracellular DNA [131,138,139,140].

Interestingly, *C. albicans* and *C. glabrata* also behave differently on different surfaces when it comes to initiating biofilm formation. Cleary, each species has a different propensity to form biofilm on a given surface. For instance, *C. albicans* is known to adhere better to latex and silicone elastomer while showing less biofilm formation on polyvinyl chloride, polyurethane, or 100% silicone [134]. *C. albicans* also adheres and forms biofilm on different surfaces of denture base materials, having higher biofilm formation on the surface of alloy and lower biofilm formation on methacrylate-based denture material [141]. Moreover, polyetherurethane treated with 6% of polyethylene oxide was found to reduce the metabolic activity of cells and the total biomass of *C. albicans* biofilms [142]. On the other hand, while all other pathogenic *Candida* species have greater biofilm formation on Teflon, *C. glabrata* prefers polyvinyl chloride to form biofilm [143]. Interestingly, other components of the environment might alter the ability of *Candida* species to adhere to a given surface. For instance, the presence of saliva has been shown to decrease the ability of *C. albicans* to form biofilm in vitro [144,145].

Depending on the antifungal drug, *C. albicans* and *C. glabrata* biofilms might be able to resist the therapeutic action of the drug. For instance, Choi and colleagues (2007) [146] measured the in vitro susceptibilities of biofilms of *C. glabrata* and *C. albicans* bloodstream isolates, showing that both biofilms were resistant to fluconazole and only moderately resistant to amphotericin B, while exposure to 0.25 to 1 µg/mL of caspofungin and micafungin lead to an 80% reduction of the biofilms. Moreover, voriconazole is also able to reduce *C. albicans* and *C. glabrata* biofilms, being present at 0.25 mg/L or being used as a surface coating at different concentrations [147]. Nevertheless, when growing on an RPMI 1640 medium, *C. glabrata* mature biofilms have shown to be less susceptible to caspofungin and anidulafungin than *C. albicans* mature biofilms on a polystyrene surface [148], showing that each species biofilms might resist differently to the same antifungal drug. Although reacting differently to antifungal drug exposure, the strategy to achieve resistance seems to be very similar between the two species. The mechanisms known to underlie resistance to antifungal drugs in *Candida* biofilms are believed to be related to alterations in the metabolic activity, the role of the extracellular matrix as a barrier for diffusion, the role of its EPS components, and the presence of persister cells within the biofilm [20,149]. Both species suffer the upregulation of drug efflux pump-encoding genes [138,150], as well as seem to rely on the β-1,3-glucans present on the extracellular matrix [151,152]. 

Biofilms also allow for the survival of *Candida* species by protecting them against the host immune system. The extracellular matrix is essential for the protection against the action of neutrophils by inhibiting the release of neutrophil extracellular traps (NETs) [153]. Moreover, the β-glucans present on the matrix avoid the activation of neutrophils, actually inhibiting the reactive oxygen response, thereby being an important distracting mechanism to evade the innate immune system [154]. Biofilms are also believed to resist well the action of the innate immune system due to its heterogenicity, given the different types of cells and different metabolic activity the cells might present on biofilms [155].

The combination of *C. albicans* and *C. glabrata* to form biofilm has been well described and the two species are usually found together in niches of candidiasis patients [60]. A recent study has shown that a ratio of *C. albicans* to *C. glabrata* of 1:3, significantly increases the total biofilm biomass comparatively to a *C. albicans* monoculture or ratio of *C. albicans* to *C. glabrata* of 1:1. This co-culture biofilm exhibited a high heterogenicity with *C. albicans* hyphae and *C. glabrata* cells clustered together in a 3D-structure. Interestingly, an upregulation of *HWP1* and *ALS3* genes is observed in this mixed-species biofilm, as well as an increased resistance to caspofungin [3]. *C. albicans* and *C. glabrata* are also known to form biofilms with bacteria from different host niches, usually relying on quorum-sensing mechanisms for the establishment of an interaction [20,155]. All the possible interactions between species increase the complexity of this vast field, pointing out the big clinical challenge of biofilm formation. 

## 4. Host Immune System Evasion

Throughout infection, when the first line of defense has been breached by invasion into deeper tissues, *Candida* pathogens have to cope with cells of the host innate immune system. Interaction with the host immune system, and the ability to overcome it, is one of the main virulence features for fungal pathogens.

At early stages of infection, upon an interaction between *Candida* pathogens and epithelial cells, the former are recognized as invasive microorganisms by Pattern Recognition Receptors (PRRs) localized at host epithelial cell surfaces. PRRs interact with Pathogen Associated Molecular Patterns (PAMPs), such as β-1,3-glucan or chitin, present on microbial cells, thereby inducing a host response [156,157]. Epithelial cells, that are part of the innate immunity, not only secrete antimicrobial peptides, such as β-defensins and LL-37 [158,159,160], to try to control fungal infection, but also release proinflammatory mediators, such as chemokines and cytokines, triggering the recruitment of phagocytic cells, such as neutrophils, macrophages, and dendritic cells. These innate immune system cells also have PRRs in their surfaces, such as the C-type lectin receptor Dectin-1 [161], allowing the recognition of the invading pathogens and thereby inducing phagocytosis [162]. After phagocytosis, dendritic cells are responsible for the link between innate and adaptative antifungal immunity, presenting the *Candida*-specific antigens to naïve T-helper cells [163]. Therefore, in an immunocompetent host, this host–*Candida* interaction ultimately leads to the elimination of the pathogen. Otherwise, in immunocompromised individuals, a persistent infection, such as chronic mucocutaneous candidiasis, candidemia and/or persistent visceral candidiasis might be established. 

Immune interaction can be translated in distinct spectrums, from avoidance of recognition by host immune cells to escaping or surviving immune attack. Masking PAMPs on the cell wall to avoid recognition, macrophage activation, and consequent phagocytosis is a common strategy of fungal pathogens during interaction with immune cells [164]. Generally, yeasts’ cell wall is composed of a carbohydrate-rich inner layer and a protein-rich outer layer of heavily mannosylated proteins and phospholipomannan [165]. The outer layer acts as a shield of immunostimulatory components of the inner layer, such as β-1,3-glucan or chitin, playing a key role in protection and evasion from immune recognition [166]. β-1,3-glucan is the main polysaccharide present in the cell wall of *C. albicans*, *C. glabrata*, and other pathogenic *Candida* species, and is a key PAMP recognized by the host immune system [167]. Recognition of β-1,3-glucan by Dectin-1 receptor prompts phagocytosis by macrophages and neutrophils [161]. 

In order to avoid immune recognition, *C. albicans* resorts to cell wall remodeling, effectively masking β-glucan from the cell surface. The first reported case of active PAMP masking by *Candida* species was reported by Ballou et al. (2016) [167]. *C. albicans* was shown to mask β-glucan in response to lactate [167], which is a relevant physiological metabolite present in *Candida* niches, such as the vaginal tract and blood, or produced by the host microbiota [168], with which *Candida* interact. Lactate-mediated β-glucan masking is modulated by a signaling pathway associated with the G-protein coupled receptor Gpr1 and the transcription factor Crz1 [167]. This pathway reduces *C. albicans* uptake by macrophages and decreases the inflammatory response (TNFα and MIP1α) and neutrophil recruitment [167]. The participation of lactate was also observed later in low oxygen environments [169]. *C. albicans* was found to mask β-glucan upon oxygen deprivation, hindering recognition by Dectin-1 of polymorphonuclear leukocytes (PMNs). This was seen to modulate PMN responses, crippling phagocytosis, action of extracellular DNA traps, and reactive oxygen species (ROS) production [169]. Interestingly, β-glucan masking was prolonged by the build-up of lactate levels produced by PMNs [169]. Later, another study reported that hypoxia promotes β-glucan masking in *C. albicans* [170]. Hypoxia-induced masking is dependent on mitochondrial function and cAMP-protein kinase A (PKA) signaling, leading to reduced macrophage phagocytosis and cytokine (IL-10, RANTES, and TNF-α) production [170]. 

Changes in carbon source result in cell wall modifications with correspondent changes in virulence and immune properties [108,171]. As mentioned before, the cell wall is a complex structure with not only glucan, but also mannans, phosphomannans, and chitin [157], although distinct *Candida* species display different glucan exposure and mannan complexity [166]. β-glucans and chitin are located in the inner-most layer, while mannans are present in the outer layer [172,173,174]. Because of such structure, mannan plays an important role in reducing immunogenic exposure of β-glucan [175], but coordinated chitin and glucan exposure has also been reported to occur in *C. albicans* [176,177,178]. Moreover, cell wall structure and mannans affect virulence in different ways in *C. albicans* and *C. glabrata* [179,180,181]. Recently, mannan structure was found to affect glucan exposure in both *C. albicans* and *C. glabrata*, albeit in distinct ways. Deletion of mannosyltransferase family genes was associated with loss of negatively-charged acid-labile mannan and less efficient glucan masking in *C. albicans* (e.g., *Δcgmnn2*), while in *C. glabrata* increased glucan exposure density was associated with mutants displaying shorter backbones (e.g., *Δcgmnn1* and *Δcganp1*) [166]. Previously, another study had shown that the β-1,6-mannosyltransferase encoded by *C. albicans MNN10* is required for backbone synthesis and influences immune recognition [182]. Absence of Mnn10 results in reduced *C. albicans* virulence, enhanced antifungal immunity by T helper cells, and increased recruitment of monocytes and neutrophils. Reinforcing the notion of a complex interplay among cell wall polysaccharydes, mannosyltransferase activity was also associated with β-1,3-glucan masking from Dectin-1 recognition and modulatory action of cytokine production by macrophages [182]. Another study showed how *C. albicans* cell wall responds to immune attacks by NETs [176]. β-glucan exposure and enhanced Dectin-1 recognition is dependent on fungal-pathogen crosstalk, as this response is dependent on neutrophil NET-mediated damage and fungal signaling cascades based on the MAP kinase Hog1. Cell wall structure in response to a neutrophil attack was found to affect more than one component, as Hog1 signaling leads to chitin deposition via the chitin synthase Chs3 and posterior cell wall remodeling via Sur7 and Phr1. Accordingly, with the enhanced immune recognition by Dectin-1 after a NET-mediated attack, macrophage cytokine response was also increased [176]. 

Much like *C. albicans*, *C. glabrata* resorts to cell wall remodeling in order to avoid the host’s immune system, although the underlying mechanisms are mostly unknown. As indicated by increased TNFα secretion and increased efficacy of pathogen killing by macrophages, deletion mutants with disturbed cell wall integrity and altered accessibility of PAMPs caused a stronger inflammatory response. *C. glabrata* deletion mutants lacking cell surface-associated proteases (yapsins) or mutants with defective protein glycosylation were related with a stronger inflammatory response by macrophages [13,181]. Nevertheless, mutations affecting mannan, but not those affecting glucan or chitin, were found to reduce the uptake of *C. glabrata* cells by murine macrophages, suggesting that mannose side chains or mannosylated proteins are ligands recognized by macrophages [183]. 

Knowledge regarding the PRRs responsible for *C. glabrata* recognition by macrophages is limited. Notwithstanding, as in *C. albicans* infections, C-type lectin receptors are thought to be involved in *C. glabrata* recognition by the host immune system. Specifically, dectin-1 and dectin-2, which recognize cell wall β-glucan, and mannan and β-glucan respectively, have been reported to be involved in the recognition of this pathogen [184,185]. 

Unlike *C. albicans* that put effort in escaping the immune system, it is hypothesized that inducing the recruitment of macrophages to the site of infection in vivo is part of the *C. glabrata* immune system evasion strategy [21]. *C. glabrata* infection did not substantially activate any MAPK pathway, including Erk1/2 (Extracellular signal-regulated kinases), SAPK/JNK (Stress-activated protein kinases/Jun amino-terminal kinases), and NF-κB signaling. Accordingly, it was found that upon infection of macrophages, the only cytokine significantly induced is GM-CSF, whereas the induction of other proinflammatory cytokines (TNF-α, IL-1β, IL-6, IL-8, and IFN-γ) is low [21,65,186]. GM-CSF is a potent activator of macrophages and induces differentiation of precursor cells as well as the recruitment of macrophages to sites of infection. This may explain the enhanced tissue infiltration of mononuclear cells, but not neutrophils, observed in vivo [186]. Considering the ability of *C. glabrata* to survive and replicate within macrophages, it is therefore speculated that persistence within macrophages is a possible strategy of immune evasion in this pathogen [21]. 

Immune evasion by pathogens also entails escaping from the complement system, another pathway of the innate immune system that facilitates phagocytosis. To evade immune response, several pathogens were shown to sequester or bind complement regulators, such as factor H (FH) [187,188,189,190]. *C. albicans* expresses the glucose transporter Hgt1 that binds FH, therefore reducing complement regulatory activity and limiting phagocytosis and killing by neutrophils [191]. SAP proteases produced by *C. albicans* not only cause tissue damage [192], but also contribute to immune evasion, as Sap2 is able to cleave antimicrobial peptides and complement proteins [193,194]. *C. albicans* Sap2 also cleaves FH and its receptors (CR3 and CR4) on macrophages, thus limiting their activation [195]. Additionally, *C. albicans* was also seen to bind yet another complement regulator (vitronectin) to modulate the hosts innate response [196]. The *C. albicans* pH-regulated protein Pra1 was recently implicated in the modulation of immune response [197,198]. It was seen to cleave the complement component C3, blocking the complement effector function and interfering with killing by neutrophils [197]. Pra1 is also implicated in adaptive immune response, as it binds to mouse CD4^+^ T cells and reduces cytokine (IFN_ϒ_ and TNF) secretion and antigen stimulation [198]. 

Ultimately, many yeast cells are engulfed by macrophages, hence survival and replication or subsequent escape remain important features. Upon phagocytosis, the phagosome carrying the ingested microorganism, fusion with a lysosome is one central antimicrobial mechanism of macrophages [164]. In the phagolysosome, fungal pathogens have to survive the harsh environment, typically characterized by carbon source limitation, production of reactive oxygen and nitrogen species, and acidification of the phagosomal compartment [199,200,201]. Mature phagolysosomes are normally strongly acidified, inducing antimicrobial effector mechanisms such as the activity of hydrolytic enzymes. Nonetheless, both *C. albicans* and *C. glabrata* are able to actively limit phagosome maturation in macrophages to prevent acidification and limit hydrolytic attack [21,202]. Environmental alkalinization by amino acid use as carbon sources, which results in ammonia extrusion, has been acknowledged as a strategy of these pathogens to actively raise phagosome pH [199,203,204]. Moreover, extracellular pH-raising triggers the yeast–hyphal switch in *C. albicans* [199,204]. Regarding carbon source availability, macrophages actively deprive pathogens of glucose, and therefore alternative carbon sources must support fungal growth. Accordingly, genes coding for enzymes of glyoxylate cycle, gluconeogenesis, and β-oxidation of fatty acids were found to be upregulated in both *C. glabrata* and *C. albicans* cells ingested by macrophages [13,18,205]. Very recently, the glyoxylate cycle gene *ICL1* was demonstrated to be crucial for the survival of *C. glabrata* in response to macrophage engulfment [206]. Disruption of *ICL1* rendered *C. glabrata* cells unable to utilize acetate, ethanol, or oleic acid and conferred a severe attenuation of virulence in the mouse model of invasive candidiasis [206]. Further, genes of the methylcitrate cycle, which is important for the degradation of fatty acid chains and which allows the use of lipids as alternative carbon sources, were also found to be upregulated in *C. glabrata* [13]. Downregulation of protein synthesis as well as upregulation of amino acid biosynthetic pathways and amino acid and ammonium transport genes are features of the nitrogen deprivation faced by fungal cells inside the macrophages [13]. 

Although being mainly known by macrophage escape, strategies to counteract oxidative and nitrosative stress have been described in *C. albicans*. For instance, flavodoxin-like proteins are part of the antioxidant response of this species by reducing ubiquinone, which acts as a membrane antioxidant [207]. Expression of four flavodoxin genes (*PST1, PST2, PST3, YCP4*) is required for *C. albicans* virulence and resist neutrophil attack [207]. Other than oxidative burst modulation, *C. albicans* was also reported to modulate the nitrosative stress exerted by macrophages [208]. Nitric oxide production is dependent on the enzyme nitric oxide synthase, which utilizes arginine as a substrate. By increasing chitin exposure, *C. albicans* induces the host arginase-1 enzyme, which competes with the nitric oxide synthase enzyme and prevents the conversion of L-arginine to nitric oxide [208]. Interestingly, the use of amino acids as carbon source is much more prominent in *C. albicans* than other fungi [199] and amino acid metabolism has been associated with more than one mechanism of phagocyte escape/survival. Arginine was also found to play a role in hyphal development upon phagocytosis by macrophages, thus contributing to escaping the phagosome [209]. Additionally, in poor glucose conditions, *C. albicans* excretes amino acid-derived ammonia that increases external pH and interferes with the acidification of the phagosome [204,210], as mentioned before. Moreover, the transcription factor Stp2 involved in amino acid acquisition, is required to prevent phagosome acidification [204,209] based on the SPS amino acid sensing system [211]. Regarding the contribution of nitrosative stress to macrophage defense against *C. glabrata*, it is known that this pathogen induces only low NO production by murine macrophages [13].

*C. glabrata* shows increased tolerance to oxidative stress when compared to other yeasts, including *Saccharomyces cerevisiae* and *C. albicans* [212], mostly associated with the activity of the catalase Cta1, the superoxide dismutases Sod1/Sod2 and the glutathione and thioredoxin pathways [212,213,214]. Despite this, it is speculated that ROS play a minor role in killing *C. glabrata* cells, since experimental inhibition of ROS production in macrophages did not result in increased fungal survival [215]. In contrast to *C. albicans*, mobilization of intracellular resources via autophagy is an important virulence factor that supports the viability of *C. glabrata* in the phagosomal compartment of innate immune cells [23]. Phagocytosis induces peroxisome production in *C. glabrata* cells, which are then degraded via pexophagy, a specialized form of autophagy [23]. 

Moreover, besides carbon and nitrogen, trace elements such as iron are important for yeast growth. Macrophage-engulfed *C. albicans* upregulate a set of genes involved in iron homeostasis, for example, the ferric reductase genes *FRE3* and *FRE7*, as well as uptake systems for other trace metals, such as copper (*CTR1*) and zinc (*ZRT2*) [18]. *C. glabrata* is also able to sense and respond to iron limitation, although it has not been shown to use host iron-binding proteins as iron sources and is unable to use heme or hemoglobin. Instead, *C. glabrata* uses the siderophore-iron transporter Sit1, which is essential for utilization of ferrichrome as an iron source under iron-deficient conditions, and for iron-dependent survival in macrophages [216,217]. This iron acquisition system improves the fitness of *C. glabrata* when it is subsequently exposed to macrophages [216]. Recently, PI3K-kinase (encoded by *VPS34*) signaling was revealed to play a central role in *C. glabrata* iron metabolism and host colonization. However, the strategies by which *C. glabrata* gains iron within macrophages remain unknown [218]. 

While *C. glabrata* is most known for a persistence strategy and survival inside macrophages, due to its high-intrinsic stress tolerance, *C. albicans* is best known for active escape via hyphal growth and phagocyte piercing. In 2014, the model of macrophage piercing due to polarized growth of hyphae was challenged by two studies [17,219]. The study by Wellington et al. (2014) [219] showed that *C. albicans* escape is not exclusively due to disruption by hyphae. The pyroptosis pathway, a proinflammatory programmed cell-death process that is dependent on caspase-1, leads to interleukin production and macrophage lysis [220] and occurs concurrently with hyphae-mediated damage. Pyroptosis has been described to occur in response to intracellular bacteria, and thus is hypothesized to achieve the goal of destroying macrophages themselves in order to eradicate phagocyted pathogens [221]. *C. albicans* yeast-to-hyphae transition induces macrophage pyroptosis, therefore indicating that phagocyte damage caused by this pathogen is a more complex mechanism than originally postulated [17,219]. Accordingly, the study by Uwamahoro et al. (2014) [17] added more evidence of this escape mechanism. The triggering of pyroptosis is necessary for full macrophage damage upon hyphal formation and is activated in early phagocytosis, followed by a more robust hyphal formation that is indeed the main mechanism of macrophage killing in a later phase [17]. 

*Candida* thrives on multiple carbon sources to survive inside macrophages, but these depend on glucose for viability. Recently, Tucey et al. (2018) [222] demonstrated that *C. albicans* exploits this limitation by depleting glucose, and triggering rapid macrophage death, in vitro. Additionally, they showed that *C. albicans* infection promotes the disruption of host glucose homeostasis in vivo and verified that glucose supplementation improves host outcomes under systemic fungal infection [222]. Thus, depriving host immune cells for glucose seems to be one mechanism of *C. albicans* to induce phagocyte cell death and actively escape from those. Moreover, it was recently discovered that candidalysin is both a central trigger for Nlrp3 inflammasome-dependent caspase-1 activation via potassium efflux, and a key driver of inflammasome-independent cytolysis of macrophages and dendritic cells upon infection with *C. albicans* [223]. This study suggests that candidalysin-induced cell damage is a third mechanism by which *C. albicans* induces phagocyte cell death in addition to damage caused by pyroptosis and the growth of glucose-consuming hyphae [223]. *C. albicans*-induced activation of the Nlrp3 inflammasome, leading to secretion of IL-1β cytokine, is a crucial myeloid cell immune response needed for antifungal host defense [224]. Very recently, Rogiers and co-workers (2019) [225] identified candidalysin as the fungal trigger for Nlrp3 inflammasome-mediated maturation and secretion of IL-1β from primary macrophages. Therefore, the expression of candidalysin is speculated to be one of the molecular mechanisms by which hyphal transformation equips *C. albicans* with its proinflammatory capacity to prompt the release of bioactive IL-1β from macrophages [225].

A more controversial evasion mechanism, based on quorum-sensing stimulation of immune recognition, has been reported in both *C. albicans* and *C. glabrata*. In the case of *C. albicans*, white cells specifically (not opaque cells) secrete E,E-Farnesol, a stimulator of macrophage chemokinesis [226]. Farnesol secretion was found to increase macrophage migration and tissue infiltration [226]. This strategy has been associated with immune evasion by the concealing of the pathogens inside immune cells themselves, an environment where *Candida* species can survive. 

Conversely to *C. albicans*, *C. glabrata* is incapable of hyphal differentiation, failing at this virulence trait. Although never observed for clinical isolates, interestingly, work by Brunke et al. (2014) [227] has shown that *C. glabrata* cells co-incubated during six months with macrophages, were able to produce pseudohyphae structures and evolve into a hypervirulent phenotype characterized by higher macrophage damage and faster escape.

**Figure 3:**
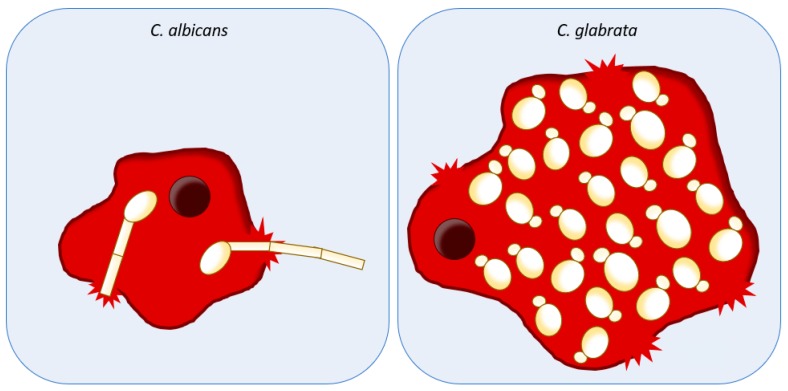
Schematic representation of *C. albicans* and *C. glabrata* host immune system evasion. *C. albicans* actively escape from host immune system cells through hyphal growth and phagocyte piercing. *C. glabrata* is most known for a persistence strategy, surviving and thriving inside macrophages, ultimately leading to immune cells lysis due to fungal load.

Overall, the two more prevalent pathogenic yeasts, *C. albicans* and *C. glabrata*, follow two main different strategies to achieve the same ultimate goal: survive host immune response. On one hand, *C. albicans* hyphal forms actively pierce the membrane of macrophages as a mechanism of killing and escape. On the other hand, *C. glabrata*, that lacks morphological plasticity, survives and replicates within macrophages due to its remarkable ability to surpass its harsh environment, ultimately leading to macrophage lysis after several days due to fungal cells overload [21] (Figure 3).

## 5. Conclusion and Perspectives

The pathogenic yeasts *C. albicans* and *C. glabrata* are the two most prevalent *Candida* species isolated from candidiasis patients worldwide, yet they are phylogenetically, genetically, and phenotypically very different. Indeed, each species displays divergent virulence traits, indicating differential adaptation to the human host. 

A greater number of virulence mechanisms has been described in *C. albicans*, most of which are not known to occur in *C. glabrata*. Yeast-to-hyphae dimorphism is one of the most striking divergent features. Hyphal morphology is associated with several key *C. albicans* traits (tissue invasion, biofilm formation, immune evasion), but is absent in *C. glabrata*. The result is that *C. glabrata* must have acquired other molecular mechanisms to reach the same goals. While tissue invasion in *C. albicans* occurs by proteolytic enzyme secretion and hyphal penetration of host tissues, *C. glabrata*-induced tissue damage is quite negligent in comparison and is thought to occur via endocytosis induction by host cells. 

A similar observation can be made regarding biofilm formation, where *C. albicans* expresses hyphal-specific adhesins and regulators required for adhesion, while *C. glabrata* biofilms are much less “bulky”. Morphological dimorphism also supports noticeable differences in immune evasion, as hyphal development and phagosome piercing is the main phagocyte escape mechanism employed by *C. albicans*, while such a strategy is absent in *C. glabrata*, which rather survives (and thrives) in the phagosome. 

Dissimilar traits between *C. albicans* and *C. glabrata* have been identified, and such attributes have been extensively studied in *C. albicans*. However, the molecular mechanisms specific to *C. glabrata* that allow such a disparate species to cause human candidiasis demand further scrutiny. Indeed, the up rise of *C. glabrata* as a key fungal pathogen can only be prevented with specific therapeutic options that match its specific virulence traits.
